# Spontaneous viral clearance of hepatitis C virus (HCV) infection among people who inject drugs (PWID) and HIV-positive men who have sex with men (HIV+ MSM): a systematic review and meta-analysis

**DOI:** 10.1186/s12879-016-1807-5

**Published:** 2016-09-05

**Authors:** Daniel J. Smith, Ashly E. Jordan, Mayu Frank, Holly Hagan

**Affiliations:** 1 Rory Meyers College of Nursing, New York University, New York, NY 10010 USA; 2 Center for Drug Use and HIV Research, New York University, New York, NY 10010 USA

**Keywords:** Hepatitis C virus, MSM, PWID, Systematic review, Meta-analysis, Spontaneous HCV clearance

## Abstract

**Background:**

Hepatitis C virus (HCV) infection causes significant morbidity and mortality among people who inject drugs (PWID) and HIV+ men who have sex with men (MSM). Characterizing spontaneous viral clearance of HCV infection among PWID and HIV+ MSM is important for assessing the burden of disease and treatment strategies in these populations.

**Methods:**

Electronic and other searches of medical literature were conducted. Reports were eligible if they presented original data from upper-middle- and high-income countries on laboratory-confirmed HCV infection and spontaneous viral clearance among PWID or HIV+ MSM. Pooled estimates of spontaneous viral clearance were generated using fixed-effect and random-effects models. Meta-regression examined potential predictors related to individual characteristics and research methodology.

**Results:**

The meta-analysis estimated that spontaneous viral clearance occurs in 24.4 % of PWID and 15.4 % of HIV+ MSM. In univariate meta-regression among PWID, male sex and age were significantly associated with spontaneous viral clearance, and in multivariate analysis, male sex and HIV positivity were predictors of spontaneous viral clearance; among HIV+ MSM no variables were found to affect spontaneous viral clearance.

**Conclusion:**

The variability in estimates of spontaneous viral clearance between HIV+ MSM and PWID suggests the impact of HIV co-infection and HCV re-infection. Due to limited data on additional factors that may affect the natural history of HCV, more research is needed to further understand spontaneous viral clearance in these risk groups.

**Protocol registration:**

The protocols for the PWID and HIV+ MSM research were registered with PROSPERO (CRD42014008805; CRD42013006462).

**Electronic supplementary material:**

The online version of this article (doi:10.1186/s12879-016-1807-5) contains supplementary material, which is available to authorized users.

## Background

Approximately 3 % of the world’s population is infected with hepatitis C virus (HCV), a blood borne infection that is almost entirely attributable to parenteral exposure via non-sterile injection equipment used in medical settings or to inject drugs [1]. After clinical or subclinical HCV infection up to 25 % of people will spontaneously clear the virus [2]. There is significant excess mortality attributable to liver-related injury in those with chronic HCV infection compared to the general population, and among those with chronic HCV infection, progression of the disease varies widely [3, 4].

Among people who inject drugs (PWID), high HCV incidence rates of 10–40 infections/100 person-years (PYs) contribute to a persistent and high population prevalence of 43–80 % [5–7]; as such, HCV is endemic among PWID [8].

Co-infection with HIV and HCV is common due to shared routes of disease transmission. HIV/HCV co-infection contributes to substantial, yet preventable, morbidity and mortality; specifically, liver disease progression is accelerated in HIV co-infected individuals [9, 10]. Co-infection with HIV and HCV is of considerable relevance to PWID as the majority of HIV-positive PWID also are infected with HCV (50–70 %) [11].

Within the HIV-positive population, sexual transmission of HCV also is a concern [12, 13]. In a recent meta-analysis, the incidence rate of HCV infection among HIV-positive men who have sex with men (HIV+ MSM) who are non-PWID was found to be 0.53/100 PYs [14]. While low, incidence of HCV in this population is expected to increase [14]. In a related meta-analysis, HCV prevalence was estimated to be 12 % among HIV+ MSM [15].

Characterizing spontaneous viral clearance of HCV infection among PWID and HIV+ MSM is important for assessing the burden of disease and the need for treatment in these populations. In this systematic review and meta-analysis, we synthesized the literature on the prevalence of spontaneous viral clearance within PWID and HIV+ MSM populations. This systematic review and meta-analysis and related simulations are conducted as part of the HCV Synthesis Project, which is funded to develop guidance and recommendations for HCV control strategies in the US [14–19].

## Methods

### Search strategy

Both electronic and manual searches for published literature were conducted. The databases of CINAHL, OVID, ProQuest, PubMed, and Web of Science were searched using the following terms: “HCV,” “hepatitis C,” “natural history,” “disease progression,” “clearance,” and “resolution.” For the HIV+ MSM group, the search string included variations of the terms “HIV,” “human immunodeficiency virus,” “AIDS,” “acquired immunodeficiency syndrome,” “men who have sex with men,” “homosexual,” and “gay.” Reports examining PWID were sought through the incorporation of the keywords “PWID,” “injection drug use,” and “intravenous drug use”. (See Additional file 1 for complete search strategies.)

Searches were refined using filters for publication date, peer-reviewed journal, and human studies. Additional literature was retrieved through manual searches of the reference lists of eligible reports, review articles, and methodological papers. The conduct and reporting of this project was guided by the Preferred Reporting Items for Systematic Reviews and Meta-Analyses (PRISMA) [20]. The protocols for the HIV+ MSM and PWID research were registered with PROSPERO (CRD42013006462; CRD42014008805) and subsequently published [16, 18].

### Inclusion and exclusion criteria

#### PWID

Reports that met the following criteria were eligible for inclusion in the review: (i) included participants with laboratory confirmed HCV infection and who reported current or previous injection drug use (hereafter referred to as PWID); (ii) presented original data on spontaneous viral clearance in a study sample comprised of at least 90 % PWID; (iii) published between January 1, 1990, and April 1, 2014; and, (iv) provided data on participants from upper-middle- or high-income countries. The condition in (iv) was based on the prevalence of the hepatitis B virus (HBV) carrier state, which is associated with lower rates of HCV clearance [21]. Across upper-middle- and high-income countries, the HBV carrier rate is less than 2 % whereas areas of high HBV endemicity are comprised of predominately lower-income countries such as Southeast Asia, Sub-Saharan Africa, and the Amazon Basin; HBV carrier rates in these regions are upwards of 8 % [22] and may be higher among those at risk of HIV or HCV. Reports were excluded if HIV or HBV co-infection was present in greater than 50 % of the PWID study sample, or if participants were receiving or previously had received HCV treatment. (Seven reports included HIV-positive PWID (range 1.8–57.1 %).)

#### HIV+ MSM

The following eligibility criteria were applied: (i) included male participants co-infected with HIV infection and with laboratory confirmed *acute* HCV infection and who reported having sex with other men (hereafter referred to as MSM); (ii) presented original data on spontaneous viral clearance in a study sample comprised of at least 90 % HIV+ MSM; (iii) published between January 1, 1996, and April 1, 2014; and, (iv) provided data on participants from upper-middle- or high-income countries. We established the condition in (iii) to account for the introduction in 1996 of highly active anti-retroviral therapy (HAART), which represented a significant change in the standard of care, and thus the natural history, of HIV infection. Accordingly, HAART may affect the disease progression of individuals co-infected with HIV and acute HCV. The restriction that participants had acute HCV allowed for the observation of the effect of HIV infection on the course of newly acquired HCV infection. The condition in (iv) follows from the assumption that health outcomes are influenced by the disparity between income-based country groups in the accessibility and completeness of coverage of HAART to HIV-positive individuals; a wide gap exists between treatment and need in low-income countries [23].

### HCV infection measure

The primary exposure of interest was acute or chronic HCV infection. The *preferred* criteria for defining acute HCV infection employed in our review was that endorsed by the European AIDS Treatment Network (NEAT) for which the criteria were seroconversion or a positive HCV RNA test following a documented negative HCV RNA or negative HCV antibody test in the previous 12 months [24]. Our *alternative* criterion for defining acute HCV infection required a statement in the report that all patients were acutely infected. Chronic infection was defined by HCV RNA positivity.

The importance of defining whether or not HCV was acute was less critical to those without HIV infection; however, in those with HIV the demonstration of acute infection (either by the preferred or alternative criteria) was relevant in asking whether HIV co-infection impacts spontaneous viral clearance. Among the reports presenting data on spontaneous viral clearance among HIV+ MSM, all participants acquired HIV infection prior to HCV infection; among PWID the sequence of disease acquisition was less clear.

### Outcome measures

The outcome of interest was the prevalence of spontaneous viral clearance. Spontaneous viral clearance was measured in cross-sectional studies as at least one negative or undetectable RNA test result and in longitudinal studies as consecutive negative or undetectable RNA test results. In some reports the definition of spontaneous viral clearance was not presented, but data were available on the outcome of interest.

### Screening and data collection

Two research assistants (RAs) screened abstracts and extracted data. The project director and the principal investigator reviewed all eligible reports and made final decisions on inclusion in the review and meta-analysis. We collected from the included reports data on the following domains: citation information; study cohort, period, and location; study design and methods; incidence and prevalence of spontaneous viral clearance; disease duration; and participant characteristics, particularly factors understood to be associated with clearance (e.g., age, sex, and HIV co-infection). For any report with missing or inconsistent data, we contacted the corresponding author for additional information or clarification. Among the set of reports examining PWID, six authors were contacted, and four fulfilled our data requests (67 %). Three of six authors provided additional data on HIV+ MSM (50 %).

### Report quality

Quality appraisal of each report included in this systematic review was based on an adapted version of the Quality In Prognosis Studies (QUIPS) tool, which was developed to assess potential biases in studies of prognostic factors [25, 26]. (The complete adapted instrument is available by request.) Each report was assigned an overall rating of *high*, *moderate*, or *low*, which indicated the extent to which the study design and analysis controlled for the influence of selection bias, misclassification, and confounding.

### Data analysis

Report-level prevalence of spontaneous viral clearance was estimated using the binomial distribution. Pooled estimates of spontaneous viral clearance were generated using both fixed-effect and random-effects models. The Cochran’s *Q* and *I*^2^ [27] measures provided assessments of heterogeneity, and random-effects meta-regression was performed to examine variability among the report-level estimates of spontaneous viral clearance.

We examined the possibility of assessing the role of previous infection with HIV on spontaneous viral clearance among PWID; however, because most studies did not specify which of HIV or HCV was acquired first, we were not able to directly examine this. Those PWID who were HIV positive and had unequivocal evidence of spontaneous viral clearance most likely represented an individual in whom the sequence of events was HCV infection, followed by spontaneous viral clearance, and subsequently HIV infection. All statistical analysis was conducted using Stata 13.1 [28].

## Results

### Meta-analysis

#### PWID

**Fig. 1:**
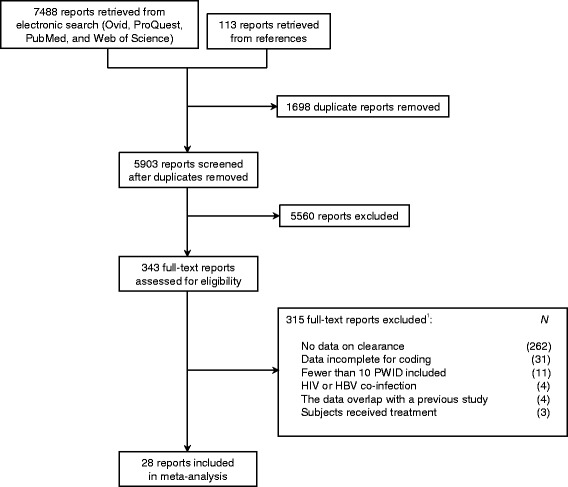
Flow diagram of the literature search and eligibility assessment for PWID. ^1^Reasons for exclusion are not mutually exclusive. A report may have been ineligible due to multiple reasons. Here, only one reason for exclusion is provided

A total of 7,488 reports were retrieved from the literature searches, and 28 were included in the final review (see Fig. 1). The reports are described in Table 1. The majority of reports described studies located in Europe (12; 43 %); the remaining samples were from the United States (7; 25 %), Australia (5; 18 %), China (2; 7 %), Canada (1; 4 %), and Iran (1; 4 %). Data on participant attributes were presented in greater than 50 % of all reports. Sixteen reports (57 %) presented age at enrollment, eighteen (64 %) provided the sex distribution, and sixteen (57 %) stated the proportion of HIV-positive individuals.

**Table 1:** Studies investigating spontaneous viral clearance of HCV among PWID

First author (pub. yr.)	Study period	Location					*N*					
			Recruitment method	Recruitment site	Definition of HCV clearance	Quality rating	Participants	Clearers	Proportion of clearance events	95 % CI		
Aberle (2006) [34]	2003–2005	Austria	Convenience sampling	Clinical setting	Methods not reported	Low	11	3	27.3	1.0	-	53.6
Aitken (2008) [35]	2005–2008	Australia	Convenience sampling	Community-based setting	1 RNA- result	Moderate	135	41	30.4	22.6	-	38.1
Alanko Blome (2014) [36]	1997–2005	Sweden	Consecutive sampling	Other setting	1 RNA- result	High	150	48	32.0	24.5	-	39.5
Boodram (2011) [37]	2002–2006	United States	Convenience sampling	Community-based setting	≥1 RNA- result over 6 months	High	113	38	33.6	24.9	-	42.3
Cournot (2004) [38]	1999–2004	France	Consecutive sampling	Clinical setting	1 RNA- result	Moderate	178	27	15.2	9.9	-	20.4
Currie (2008) [39]	1997–2007	United States	Convenience sampling	Clinical and drug treatment settings	≥2 consecutive RNA- results	Moderate	215	29	13.5	8.9	-	18.1
Dolan (2010) [40]	2005–2007	Australia	Convenience sampling	Correctional setting	1 RNA- result	Moderate	16	6	37.5	13.8	-	61.2
Garten (2008) [41]	1999–2008	China	Unspecified sampling	Clinical setting	1 RNA- result	Low	347	30	8.6	5.7	-	11.6
Gerlach (2003) [42]	1993–2003	Germany	Consecutive sampling	Clinical setting	≥1 RNA- result over 6 months	High	15	5	33.3	9.5	-	57.2
Gjeruldsen (2003) [43]	1997–1999	Norway	Consecutive sampling	Clinical setting	1 RNA- result	Moderate	50	8	16.0	5.8	-	26.2
Grebely (2007) [44]	1992–2005	Canada	Convenience sampling	Community-based setting	≥1 RNA- result	High	431	91	21.1	17.3	-	25.0
Hallinan (2007) [45]	2002–2005	Australia	Consecutive sampling	Drug treatment setting	1 RNA- result	Moderate	145	43	29.7	22.2	-	37.1
Hsieh (2014) [46]	2008–2010	China	Unspecified sampling	Correctional setting	Methods not reported	Low	513	99	19.3	15.9	-	22.7
Jauncey (2004) [47]	1992–2002	Australia	Consecutive sampling	Clinical setting	≥2 consecutive RNA- results	High	57	24	42.1	29.3	-	54.9
Keating (2005) [48]	1997–2001	Ireland	Consecutive sampling	Drug treatment setting	2 consecutive RNA- results separated by at least 12 months	High	496	191	38.5	34.2	-	42.8
Kielland (2013) [49]	1970–2008	Norway	Consecutive sampling	Drug treatment setting	1 RNA- result	Moderate	523	195	37.3	33.1	-	41.4
Lidman (2009) [50]	2004–2006	Sweden	Consecutive sampling	Clinical setting	1 RNA- result	Moderate	268	61	22.8	17.7	-	27.8
Mattsson (1993) [51]	1991–1993	Sweden	Consecutive sampling	Population-based setting	1 RNA- result	Moderate	12	4	33.3	6.7	-	60.0
Meyer (2007) [52]	2002–2007	Germany	Consecutive sampling	Correctional setting	≥1 RNA- result	Moderate	90	23	25.6	16.5	-	34.6
Osburn (2010) [53]	1997–2007	United States	Convenience sampling	Clinical, drug treatment, and community-based settings	≥1 RNA- result over 2 months	Moderate	113	31	27.4	19.2	-	35.7
Ostapowicz (1999) [54]	1990–1999	Australia	Unspecified sampling	Clinical setting	1 RNA- result	Moderate	142	2	1.4	−0.5	-	3.3
Page (2013) [55]	2000–2011	United States	Convenience sampling	Unspecified	≥2 RNA- results	High	109	26	23.9	15.9	-	31.9
Poustchi (2011) [56]	2004–2008	Iran	Other systematic	Clinical and research setting	1 RNA- result after 6 months	Moderate	28	4	14.3	1.3	-	27.2
Santantonio (2006) [57]	1999–2004	Italy	Unspecified sampling	Clinical setting	≥1 RNA- result w/in 6 months and ≥ 1 RNA- result for additional 6 months	Moderate	71	31	43.7	32.1	-	55.2
Shah (2012) [58]	2004–2007	United States	Convenience sampling	Community-based setting	1 RNA- result	Moderate	272	43	15.8	11.5	-	20.1
Thomas (2000) [59]	1988–1998	United States	Convenience sampling	Community-based organization	2 consecutive RNA- results separated by at least 5 months	High	919	90	9.8	7.9	-	11.7
van den Berg (2011) [60]	1985–2005	The Netherlands	Convenience sampling	Clinical and drug treatment settings	2 consecutive RNA- results separated by at least 4 months	Moderate	106	35	33.0	24.1	-	42.0
Wang (2007) [61]	2003–2005	United States	Unspecified sampling	Clinical and research setting	2 consecutive RNA- results	High	44	8	18.2	6.8	-	29.6

Fixed-effect meta-analysis estimate of the prevalence of spontaneous viral clearance (28 studies): 15.1 % (95 % CI 14.2, 16.0)

Random-effects meta-analysis estimate of the prevalence of spontaneous viral clearance (28 studies): 24.3 % (95 % CI 19.5, 29.1)

Heterogeneity: *Q =* 638.51, *p* < 0.001; *I* ^2^ *=* 95.8 %

**Fig. 2:**
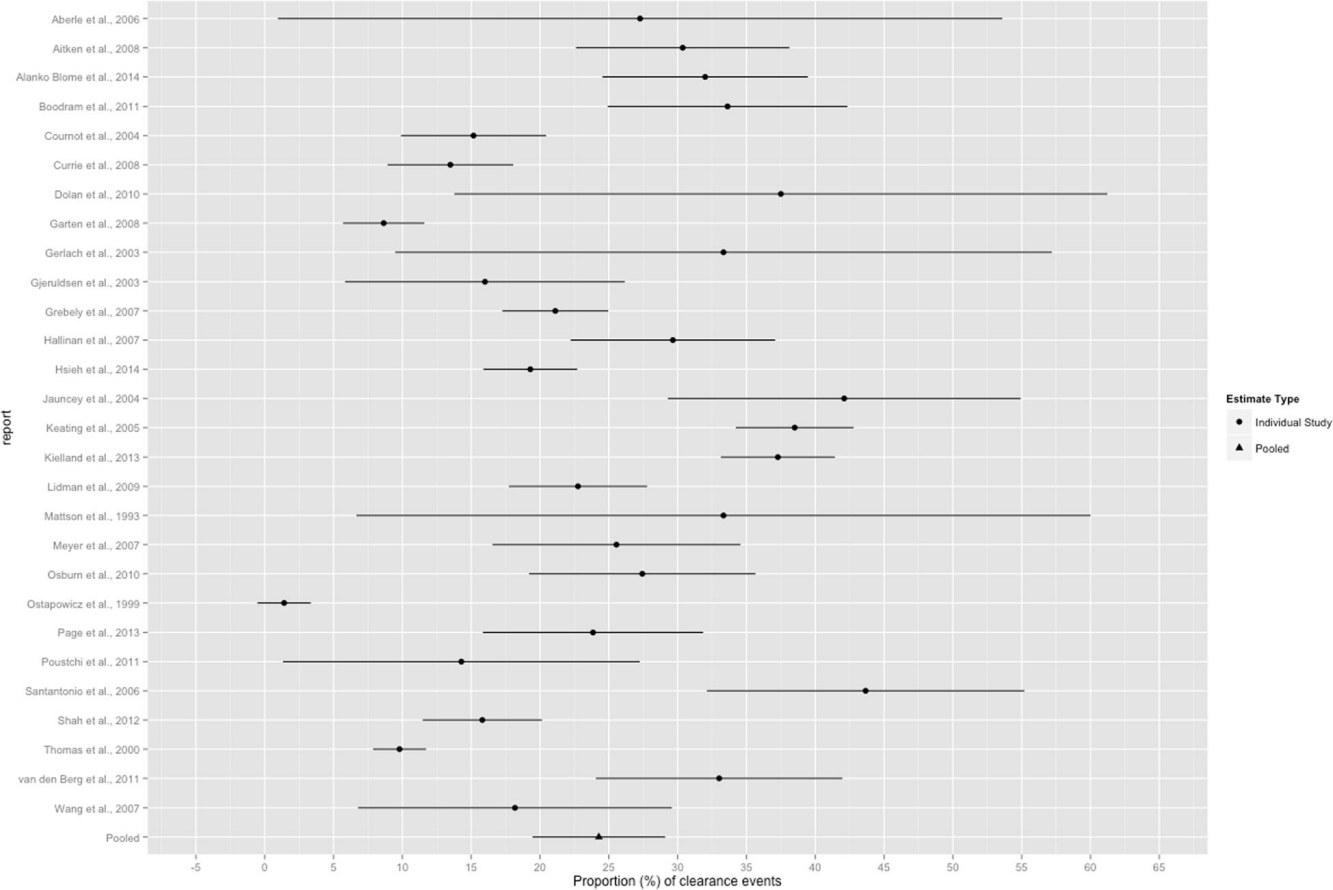
Forest plot of the estimated prevalence and 95 % CI of HCV clearance among PWID for each study

All 28 reports were included in the meta-analysis. Among 5,569 PWID, whose mean age at enrollment was 29.6 years (median 27.4; 15 reports), spontaneous viral clearance was observed in 1,236 participants. The random-effects meta-analysis estimate of the prevalence of spontaneous viral clearance was 24.3 % (95 % CI 19.5, 29.1; *Q* = 638.51, *p <* 0.001; *I*^*2*^ = 95.8 %). The estimate from each study is presented in the forest plot in Fig. 2.

Pooled subgroup estimates also were generated to account for HIV status and quality rating. Within the sets of reports for which there were data on the HIV status of PWID, spontaneous viral clearance occurred in 25.7 % (95 % CI 16.4, 35.0; 13 reports) of HIV-negative participants and in 16.1 % (95 % CI 12.5, 19.6; 3 reports) of HIV-positive participants. In stratifying the reports by quality rating, as depicted in Table 3, the prevalence of spontaneous viral clearance was 27.6, 24.1, and 15.4 % among high-, moderate-, and low-quality reports, respectively.

#### HIV+ MSM

**Fig. 3:**
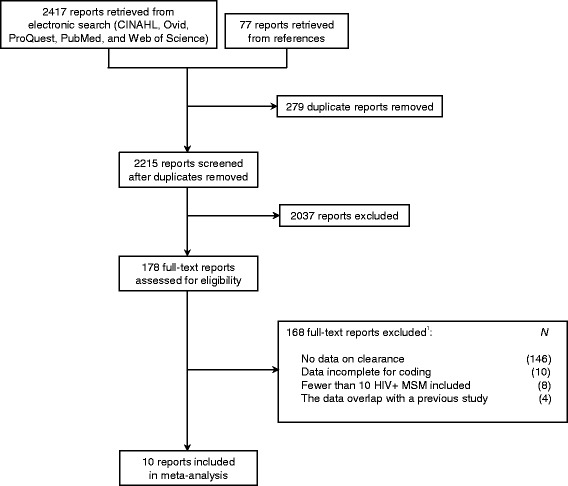
Flow diagram of the literature search and eligibility assessment for HIV+ MSM. ^1^Reasons for exclusion are not mutually exclusive. A report may have been ineligible due to multiple reasons. Here, only one reason for exclusion is provided

Following from Fig. 3, the literature searches yielded 2,417 reports, of which 10 were included in the present analysis; these reports are detailed in Table 2. Seven of the reports (70 %) were on studies with cohorts drawn only from Europe. One report (10 %) examined a sample from the United States, and another report (10 %) assessed samples from North America, Australia, and Europe. The study location was not described in one report. Few participant characteristics were summarized consistently across the reports. Age at enrollment was presented in five reports (50 %), proportion on HAART in three reports (30 %), and duration of HIV infection in one report (10 %).

**Table 2:** Studies investigating spontaneous viral clearance of HCV among HIV+ MSM

First author (pub. yr.)	Study period	Location					*N*					
			Recruitment method	Recruitment site	Definition of HCV clearance	Quality rating	Participants	Clearers	Proportion of clearance events	95 % CI		
Bottieau (2010) [62]	2001–2009	Belgium	Consecutive sampling	Clinical setting	≥1 RNA- within 6 months	Moderate	65	7	10.8	3.2	-	18.3
Dietz (2012) [63]	Not reported	Germany	Not reported	Not reported	Methods not reported	Low	47	4	8.5	0.5	-	16.5
Fierer(2014) [64]	Not reported	United States	Not reported	Not reported	≥1 RNA- within 3 months	Moderate	41	5	12.2	2.2	-	22.2
Fletcher (2003) [65]	2002–2003	United Kingdom	Not reported	Clinical setting	≥1 RNA- result	Moderate	16	6	37.5	13.8	-	61.2
Gilleece (2005) [66]	1997–2003	United Kingdom	Not reported	Clinical setting	>1 RNA- result within 3 months	Moderate	50	12	24.0	12.2	-	35.8
Grebely (2014) [29]	1985–2010	Multiple locations	Not reported	Clinical, community-based, and correctional settings	2 consecutive RNA- results separated by at least 1 month	Moderate	11	0	—	—		
Martin (2013) [67]	2004–2014	United Kingdom	Consecutive sampling	Clinical setting	2 RNA- results after 6 months	High	145	31	21.4	14.7	-	28.1
Piroth (2010) [68]	2008–2009	France	Consecutive sampling	Clinical setting	≥1 RNA-	High	53	8	15.1	5.5	-	24.7
Sasadeusz (2011) [69]	2003–2007	Not reported	Consecutive sampling	Clinical setting	2 consecutive RNA- results separated by at least 3 months	Low	61	9	14.8	5.9	-	23.7
Thomson (2011) [70]	2005–2009	United Kingdom	Not reported	Clinical setting	2 consecutive RNA- results separated by at least 3 months	Moderate	99	14	14.1	7.3	-	21.0

Fixed-effect meta-analysis estimate of the prevalence of spontaneous viral clearance (9 studies): 15.2 % (95 % CI 12.3, 18.1)

Random-effects meta-analysis estimate of the prevalence of spontaneous viral clearance (9 studies): 15.4 % (95 % CI 11.5, 19.3)

Heterogeneity: *Q* = 13.29, *p* = 0.102; *I* ^2^ = 39.8 %

Only 9 of the 10 reports were included in the meta-analysis. One report provided a count of zero spontaneous clearance events in the sample. We chose the conservative approach to handling the zero-count issue, which was to exclude the report from the pooled analysis.

**Fig. 4:**
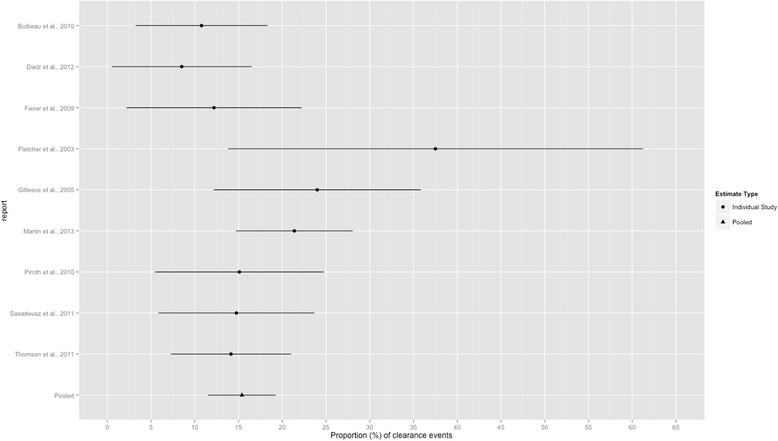
Forest plot of the estimated prevalence and 95 % CI of HCV clearance among HIV+ MSM for each study

In aggregate, there were 588 HIV+ MSM with a mean age of 40.0 years (median 40.7; 4 reports); 96 participants experienced spontaneous viral clearance. The random-effects meta-analysis estimate of the prevalence of spontaneous viral clearance was 15.4 % (95 % CI 11.5, 19.3; *Q* = 13.29, *p =* 0.102; *I*^*2*^ = 39.8 %). The forest plot in Fig. 4 provides the estimates from the contributing reports. Subgroup estimates based on quality rating also were obtained. As shown in Table 3, among high-, moderate-, and low-quality reports, prevalence of spontaneous viral clearance was 19.2, 15.8, and 11.3 %, respectively.

**Table 3:** Prevalence of spontaneous viral clearance among PWID and HIV+ MSM stratified by quality rating

	PWID		HIV+ MSM	
Quality rating	Estimate	No. of reports	Estimate	No. of reports
High	27.6	9	19.2	2
Moderate	24.1	16	15.8	5
Low	15.4	3	11.3	2

### Meta-regression

To examine the effect of report and participant characteristics on the prevalence of spontaneous viral clearance, we conducted random-effects meta-regression. Because covariates hypothesized to contribute to spontaneous viral clearance were not consistently collected or reported across reports, our meta-regression was limited to a small set of factors. Both univariate and multivariate meta-regression were performed.

#### PWID

Only two variables were significant in univariate analysis: male sex and age (*p* < 0.05). Spontaneous viral clearance was lower in both males and older individuals. We examined the relationship among pairs of variables (specifically, male sex, HIV-positivity, and age) through contingency tables. Fisher’s exact tests revealed no significant associations between factor dyads (results not shown; see Additional file 2). Of particular interest to our study was the impact of HIV. In our preferred multivariate model, presented in Table 4, male sex (*p* < 0.021) and HIV positivity (*p* < 0.036) were significant predictors.

**Table 4:** Random-effects meta-regression results for spontaneous viral clearance among PWID

Factor	β	SE	*p*	95 % CI		
HIV-positive (%)	−0.253	0.105	0.036	−0.487	-	−0.020
Males (%)	−0.414	0.151	0.021	−0.750	-	−0.078
Constant	59.638	11.252	0.000	34.567	-	84.710
No. of reports	13					

#### HIV+ MSM

Neither univariate nor multivariate analysis provided any evidence of an effect on spontaneous viral clearance of any of the variables (i.e., age, proportion on highly active antiretroviral therapy, recruitment site, and quality rating) evaluated.

## Discussion

In this systematic review and meta-analysis, we estimated that the prevalence of spontaneous viral clearance is 24.4 % in PWID and 15.4 % in HIV+ MSM. Although the estimates for PWID and HIV+ MSM were not directly compared here, the difference may be related to the impact of HIV co-infection on the natural history of HCV. The rates of spontaneous viral clearance were similar in HIV+ MSM (15.4 %) and PWID with HIV infection at the time that HCV clearance was evaluated (16.1 %). Our estimate of 24.4 % among PWID was very closely similar to the estimate from a pooled analysis of clearance among 632 participants in multiple studies (25 %) who were observed following acute infection [29].

Among HIV-positive PWID, lower clearance cannot reliably be attributed to the effect of HIV infection on viral kinetics because the temporal relation between HIV and HCV infections in these individuals is unclear. Moreover, estimates of spontaneous viral clearance from most studies do not necessarily represent true rates but rather the prevalence of cleared infection. Evidence of clearance at any given time in an individual is the cumulative result of behaviors that led to infection events, including multiple re-infections, and the host and viral characteristics that govern response to acute HCV infection. Although re-infection post-SVR is higher among HIV + MSM than among PWID, it cannot be concluded that the rates of HCV re-infection in treated and untreated HIV + MSM are higher than among treated and untreated PWID because studies of re-infection in treated PWID have in many cases excluded active injectors [14, 30]. There is no research comparing the frequency of HCV transmission behavior between these groups, and thus, there is insufficient evidence to attribute differences in clearance to differences in behavioral risk.

Our finding that female gender was associated with higher proportions of spontaneous viral clearance events is consistent with published literature demonstrating that females are more likely to clear HCV than their male counterparts in a variety of settings and other patient groups [31, 32].

Evaluation of the report-level spontaneous viral clearance data for each of the populations indicates that the meta-analysis estimates were affected by notable degrees of heterogeneity. Among PWID, both male sex and age were associated with lower proportions of spontaneous viral clearance events. In examining spontaneous viral clearance estimates in HIV+ MSM, the results did not suggest the effect of any of the factors considered. Given the null findings in univariate meta-regression on quality rating (*p* = 0.163) and study design (*p* = 0.182), and noting the low volume of reports for which we extracted data on participant characteristics, heterogeneity was most likely due to unmeasured clinical, patient-level characteristics (e.g., ethnicity, genotype, other viral infections) rather than methodological characteristics. However, our analysis of sources of heterogeneity was limited by the dearth of information about time to event, viral factors, and other participant attributes.

### Limitations

There are limitations to the meta-analysis that should be considered. One critical point is that the results of this review suggest, but do not explain, the mechanisms that lead to spontaneous viral clearance. Another important consideration is that the study methods and data provided by some of the contributing reports to this review presented challenges to the characterization of clearance in PWID and HIV+ MSM. We discuss here the main issues related to the contributing reports.

First, the samples of PWID examined in the included reports were not composed solely of individuals with a single HCV infection event, and, therefore, the estimates likely represented clearance in relation to cumulative HCV exposures via ongoing injection risk behavior [33]. Indeed, among PWID, rates of re-infection following spontaneous viral clearance are as high as 47 cases per 100 person-years [2]. Additionally, low rates of clearance observed among HIV+ PWID may represent frequent risk behavior that led to HIV infection and HCV re-infection. The high heterogeneity in the clearance estimates for both PWID and HIV+ MSM may in fact represent variability in the number of HCV infection events, in addition to genetic and other factors.

Second, the cross-sectional study design of most of the reports also limits interpreting the estimates vis-à-vis the underlying process.

Third, and related to the previous point, the definition of spontaneous viral clearance also was not uniform across studies. In particular, the criteria for spontaneous viral clearance events were notably different between cross-sectional and longitudinal reports.

## Conclusion

This systematic review and meta-analysis suggests that the prevalence of spontaneous viral clearance is higher among PWID compared to HIV+ MSM. Our findings also showed that, among PWID, male sex and HIV co-infection are negatively correlated with clearance. The data we presented are useful for modeling future morbidity, mortality, and costs related to HCV infection. Improved research methodology and examination of individual characteristics in future studies would help to determine the natural course of HCV among the high-risk populations of PWID and HIV+ MSM, and appropriate allocation of resources for HCV treatment.

## Additional files

Additional file 1: Search strategies by risk group. Provides the search strings used to locate literature across the electronic databases for both the PWID and HIV+ MSM risk groups. (PDF 6 kb) Additional file 2: Fisher’s exact tests for variables associated with spontaneous viral clearance among PWID. Provides the results of Fisher’s exact tests for variables associated with spontaneous viral clearance among the PWID samples. (PDF 7 kb)

## Abbreviations

CI: Confidence interval
HCV: Hepatitis C virus
HIV: Human immunodeficiency virus
HIV+ MSM: Human immunodeficiency virus-positive men who have sex with men
PWID: People who inject drugs
SE: Standard error
