# Supplementary material for: Spontaneous viral clearance of hepatitis C virus (HCV) infection among people who inject drugs (PWID) and HIV-positive men who have sex with men (HIV+ MSM): a systematic review and meta-analysis
Source: BMC Infect Dis. 2016 Sep 5;16(1):471. doi: 10.1186/s12879-016-1807-5 (PMC5011802; doi:10.1186/s12879-016-1807-5)
Supplement: Additional file 1: — Search strategies by risk group. Provides the search strings used to locate literature across the electronic databases for both the PWID and HIV+ MSM risk groups. (PDF 6 kb) [file 12879_2016_1807_MOESM1_ESM.pdf]

Additional file 1. Search strategies by risk group.

PWID

Ovid, ProQuest, and PubMed queries:
((HCV OR “Hep C” OR Hepc OR “Hepatitis C” OR Hepacivirus OR “hepatitis non a non b”) AND (“natural history” OR prognosis OR disease progression OR disease duration OR morbidity OR mortality OR survival) AND (PWID OR IDU OR IVDU OR “drug use” OR "injection drug use" OR "injection drug users" OR "intravenous drug use" OR "Substance Abuse, Intravenous" OR "people who inject drugs"))

HIV+ MSM

CINAHL, Ovid, ProQuest, PubMed, and Web of Science queries:
((HCV OR “Hep C” OR Hepc OR “Hepatitis C” OR Hepacivirus OR “hepatitis non a non b”) AND (“natural history” OR prognosis OR “disease progression” OR “disease duration” OR morbidity OR mortality OR survival OR resolution OR clearance OR fibrosis OR cirrhosis OR “hepatocellular carcinoma” OR HCC OR “end stage liver disease” OR ESLD) AND (msm OR "men who have sex with men" OR "men having sex with men" OR homosexual* OR gay OR bisexual* OR transsexual* OR transexual* OR transgender* OR "down low" OR "down-low") AND (HIV OR “HIV infections” OR “human immunodeficiency virus” OR AIDS OR “acquired immunodeficiency syndrome”))
