# Supplementary material for: Spontaneous viral clearance of hepatitis C virus (HCV) infection among people who inject drugs (PWID) and HIV-positive men who have sex with men (HIV+ MSM): a systematic review and meta-analysis
Source: BMC Infect Dis. 2016 Sep 5;16(1):471. doi: 10.1186/s12879-016-1807-5 (PMC5011802; doi:10.1186/s12879-016-1807-5)
Supplement: Additional file 2: — Fisher’s exact tests for variables associated with spontaneous viral clearance among PWID. Provides the results of Fisher’s exact tests for variables associated with spontaneous viral clearance among the PWID samples. (PDF 7 kb) [file 12879_2016_1807_MOESM2_ESM.pdf]

Additional file 2. Fisher's exact tests for variables associated with spontaneous viral clearance among PWID.

Table 1. 2 x 2 table: The proportion of males and the proportion of HIV+ PWID.

	Proportion (%) of HIV+ PWID		
**Proportion (%) of males**	**0-25**	**26-50**	**Total**
0-50	1	0	1
51-100	9	3	12
Total	10	3	13

Fisher's exact = 1.000

1-sided Fisher's exact = 0.769

Table 2. 3 x 2 table: The proportion of males and the proportion of HIV+ PWID.

	Proportion (%) of HIV+ PWID		
**Proportion (%) of males**	**0-25**	**26-50**	**Total**
26-50	1	0	1
51-75	7	1	8
76-100	2	2	4
Total	10	3	13

Fisher's exact = 0.413

Table 3. 2 x 2 table: The proportion of males and age.

	Age		
**Proportion (%) of males**	**Below 30 years**	**Above 30 years**	**Total**
0-50	0	1	1
51-100	4	9	13
Total	4	10	14

Fisher's exact = 1.000

1-sided Fisher's exact = 0.714

Table 4. 3 x 2 table: The proportion of males and age.

	Age		
**Proportion (%) of males**	**Below 30 years**	**Above 30 years**	**Total**
26-50	0	1	1
51-75	3	7	10
76-100	1	2	3
Total	4	10	14

Fisher's exact = 1.000 0.413

Table 5. 3 x 3 table: The proportion of males and age.

	Age			
**Proportion (%) of males**	**18-28 years**	**29-39 years**	**40-50 years**	**Total**
26-50	1	0	0	1
51-75	7	3	0	10
76-100	2	0	1	3
Total	10	3	1	14

Fisher's exact = 0.520
